# Talks about Food

**Published:** 1892-03-12

**Authors:** 


### Talks about Food.

(Continued from page 36.)

### III.—HOW THE RICH LIVE.

Eating and drinking, declared a brilliant talker once, constitute three-quarters of the pleasure of life. And though this proposition was received with indignation by some of his hearers, and with somewhat contemptuous amusement by others, he still stuck to it stoutly. Probably he was no more than half in earnest; but, nevertheless, it can hardly be denied that there was a good deal to be said on his side of the question, for, when one comes to think of it, the pleasure of gratifying a healthy appetite, even if that pleasure be small, comes very frequently, and is concerned with a very considerable portion of our waking hours.

It is quite worth our while, then, to ask ourselves now and again whether our food supplies us with the maximum amount of pleasure and of health. For of this we may be sure, unless a certain diet be a health-giving one, it will fail in the end in pleasure-giving capacity also.

Now, while the diet of the poor is often lacking in both health and pleasure-giving, by reason of its insufficient quantity, its sameness, and its lack of nutritiveness, the diet of the rich errs by excess rather than by defect. Many a rich man hardly knows what the pleasure of gratifying a healthy hunger means—and this, after all, is the only kind of pleasure with regard to diet which it is wise to indulge in, unless, perhaps, just once in a way. He sits down to dinner to gratify, not his appetite, but his taste, setting to work before he is really hungry, and going on long after he is really satisfied, by the aid of various little devices to tickle the palate, and overload the jaded stomach. No doubt the "pleasures of the table," as generally understood, include this kind of pleasure, to which his cook knows well how to pander; but it is not a healthy pleasure, and in the end he will generally have to pay for it—nay, he will often find to his consternation that it will slip from his grasp altogether. In our last article we quoted a well-known authority's declaration that excess of food was responsible for far more evil than was deficiency of food; and any doctor could make out, from his own experience, a formidable list of diseases and derangements of the system consequent upon over-eating. Thus, if over-eating gives pleasure at the time, its after-effects are anything but pleasurable; and even putting after-effects out of the question, we do seriously believe that the man who regularly goes in for the pleasures of the table does not really enjoy himself a bit better than the man who lives more simply. He spends more—that goes without saying, and his pleasure may, perhaps, be of a more conscious kind; but after all, the gratification of appetite is greater than the gratification of taste, being the gratification of a healthy and natural instinct. Besides, a well-cooked and tasty dish gives five times as much pleasure when it stands alone, as when it forms one of a crowd. Who has not experienced the truth of this, on going from a grand house, where elaborate dinners are given, to a more simple house, where, as your host says, you "see your dinner before you?" You have time to think of it, to enjoy it, and to "come again"; and all this gives you just as much pleasure as nibbling at one thing after another, just to please the insatiable curiosity of your tongue.

To our thinking there is something humiliating in the reflection that we cannot be trusted to use the good things of the earth without abusing them. The upper classes have, indeed, got over the habit, for the most part, of indulging too freely in alcoholic drinks; but gluttony is a real vice, though it may not be such a serious one as drunkenness; and anything which tends to impair the health and activity of our bodies and minds is a sin against the God who made us. It would be an excellent thing if well-to-do people would take the trouble, once in a way, to find out what is the amount of food they take in the day, and what proportion of nitrogenous elements it contains. For here again we often go sadly astray. Many persons talk and act as though meat were the only food that had any real strength in it; others eat a large quantity of it because they like it, or have got into the habit of taking it; and both classes pay little or no regard to the suitability of such a diet to the kind of life they are leading. Much harm is done in this way, as any doctor can tell us; for the system becomes loaded with nitrogenous products, which are not needed for the support of the body, and thus gouty and rheumatic affections, not to mention other disorders, are caused. Now it would be no such difficult thing to find out what is the average amount of food we consume in the day, and what proportion its nitrogenous bear to its carbonaceous elements; and then to take counsel with the doctor, or to look up the subject in some practical manual, in order to see whether we are anything like right. It is by no means hard to arrive at an approximate idea as to the diet we require. Roughly speaking, we may say that a man engaged in laborious work should consume from forty-two to forty-six ounces of solid food; and for one who leads a sedentary life considerably less—say, something over thirty ounces, should suffice. Animal food should not form more than one-fourth of our diet, and where little exercise is taken, it may with advantage be less.

It may not be a very palatable doctrine, but we do believe that many a rich man would find himself much more healthy, and equally happy, or rather, more so, if he would study this question a little, regulate his diet accordingly, part with his "good cook," and replace her with one who was good in the real sense of the term—able to cook his food tastefully and well, but not addicted to all kinds of devices for making him eat more than he wants. He will find the advantage of this even more as old age creeps on—a time when superfluous food only irritates and clogs the system, instead of being carried off in violent exercise, as youth knows how to do; and he need be under no apprehension lest a simpler style of living should hurt young Tom and young Harry, who are just growing up. He himself probably began life in a very quiet way, having to work his way up in the world, like most men who are worth anything in it; and there is no reason why Tom and Harry should live upon the fat of the land, just because he happens to have money enough to supply them with it—indeed, there is every reason why they should not. We have seen a young man of thirty with dull eyes, slow, heavy, listless movements, and a corpulence of figure such as would have suited a man of advanced years, and all for no apparent reason but that his father had made a pile of money, and that he (the son) had always been used to high living, and to taking things easy.

"The principal thing we have to guard young people against," says the writer of an excellent manual on diet, "is overloading the stomach, otherwise it becomes larger from habitual distension than is appropriate for the size of the trunk, and there is in after life a tendency to gastric flatulence." For this reason, he urges, that a growing lad's meals should be sufficiently frequent to avoid any gorging, but goes on to recommend that luncheon should consist of but one dish—a Cornish pasty, bread and cheese and lettuce, or better still, a dish of roast potatoes, well buttered and peppered, and washed down with a draught of milk. We must not forget, indeed, that the growing boy or girl needs more food, and generally a larger proportion of meat, than does an adult advanced in years; but with a little care as to the respective values of the food chosen, and to its simplicity and goodness, parents might often feed their children far less expensively, and at the same time far better, than they do.

